# Platforms for studying cell–cell recognition by immune cells

**DOI:** 10.1111/imcb.70036

**Published:** 2025-05-29

**Authors:** Jordan Kramer, P Anton van der Merwe, Omer Dushek

**Affiliations:** ^1^ Sir William Dunn School of Pathology University of Oxford Oxford UK

**Keywords:** cell–cell recognition, experimental platforms, receptor/ligand interactions, signal integration, surface ligand presentation

## Abstract

Immune cells interact directly with other cells and make decisions by integrating information from many different receptor–ligand interactions at these cell–cell interfaces. Since they encounter a huge variety of normal and abnormal cells, they experience many different combinations and concentrations of ligands. Understanding immune responses therefore requires platforms that enable ligands to be easily manipulated. We review and compare the available platforms, focusing on T‐cell recognition. Although genetically modified antigen‐presenting cells (APCs) offer the most physiological system, manipulating their ligands is difficult and slow. In contrast, solid surfaces or supported lipid bilayers allow easy manipulation of ligands but lack the biophysical properties of cells, such as softness, a glycocalyx, and/or ligand mobility. A recently developed CombiCell system enables easy manipulation of ligands while conserving key biophysical properties. By comparing the advantages and limitations of each platform, we provide a framework to choose the most suitable system to study signal integration in both basic and translational contexts.

## Graphical abstract

This review compares different platforms for studying how immune cells integrate signals from co‐signaling receptor‐ligand interactions at cell–cell interfaces. By understanding the strengths and weaknesses of each platform, including which factors can be controlled by the user, immunologists can identify the most appropriate platform to address their hypothesis.

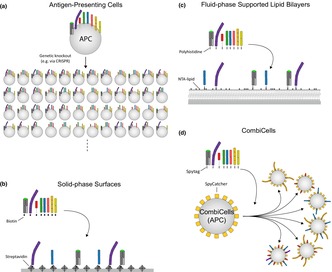

## INTRODUCTION

Multiple tissues and physiological systems communicate through direct cell–cell contacts.^1^ These interactions involve the binding of cell surface receptors to counter‐receptors or ligands, providing essential information for processes such as growth, proliferation, activation, differentiation, and migration. The immune system heavily relies on direct cell–cell contact, where immune cells interact with each other and with nearly all other cells in the body to identify and eliminate infected or otherwise abnormal cells, including cancer. Within these cellular interfaces, signals from various receptor‐ligand interactions are integrated to determine cellular responses.^2^, ^3^ Given their functional importance and accessibility to therapeutic antibodies, these interfaces are now routinely targeted clinically to, for example, redirect immune cells to attack abnormal cells.^4^, ^5^, ^6^, ^7^ Enhancing our fundamental understanding of cell–cell recognition and signal integration at cell–cell interfaces will undoubtedly improve our ability to exploit these interactions for therapeutic purposes.

**Figure 1:**
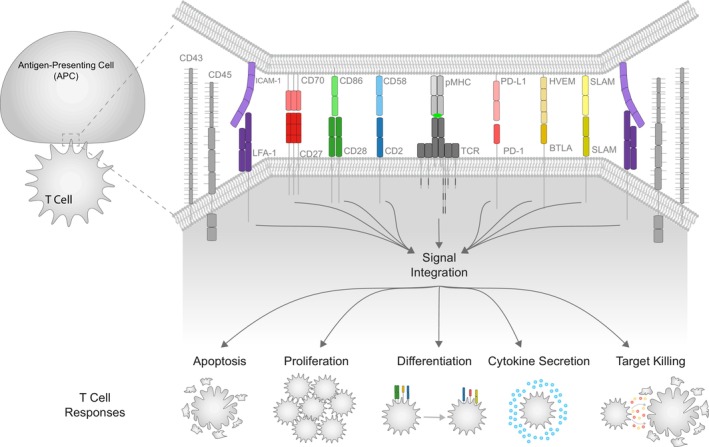
Immune cell decisions are predicated on signal integration by receptor/ligand interactions at cell–cell interfaces.

Arguably the most intensively studied cell–cell interactions are those involving T cells, which are capable of recognizing abnormal cells in nearly all tissues. T cells achieve this by using their T‐cell receptors (TCRs) to recognize ligands (antigens), often in the form of short peptides presented on major histocompatibility complexes (pMHCs). When T cells detect non‐self pMHC antigens derived from pathogens or cancers, they can initiate immune responses aimed at eliminating these threats. However, it is now clear that, in addition to the TCR, there are many other co‐signaling receptors that can significantly modify the T‐cell response (Figure 1). Co‐stimulation receptors, such as CD2, CD28, and 4‐1BB, amplify the T‐cell response, while coinhibitory receptors, such as PD‐1, BTLA, and LAG3, can attenuate or even abolish it. Collectively, these co‐signaling receptors have been implicated in the T‐cell response to infection,^8^ cancer,^9^ autoimmunity^10^ and transplantation.^11^ For example, CD58, which is the ligand for the co‐stimulation receptor CD2, is often mutated or lost on cancer cells^12^, ^13^ and human cytomegalovirus is known to reduce CD58 surface levels to evade T‐cell immunity.^14^ Beyond T cells, the concept of signal integration is believed to be fundamental to the decision‐making processes of many immune cells, including B cells,^15^ NK cells^2^ and macrophages.^16^ Consequently, there is considerable interest in understanding the mechanisms of signal integration.

The process of cell–cell recognition is also routinely exploited in clinical treatments for cancer and autoimmune diseases. A prominent example is the redirection of T cells, macrophages, or NK cells using synthetic chimeric antigen receptors (CARs) to target almost any surface antigen.^7^ Similarly, soluble bispecific T‐cell engagers target the TCR‐CD3 complex on T cells to either pMHC antigens (e.g. ImmTAX) or other antigens (e.g. BiTE) on cancer cells.^4^ Collectively, these synthetic molecules compel immune cells to form interfaces with cells they would otherwise ignore. These therapies have achieved remarkable success with several clinical approvals and many others in clinical trials. However, a major limitation is the potential for patient relapse when cancer cells emerge with reduced antigen expression and/or diminished or absent ligands for co‐signaling receptors.^17^ As a result, there is significant interest in understanding how the concentration and combination of antigens and ligands for co‐signaling receptors influence cell–cell recognition mediated by therapeutic agents.

Given the critical role of signal integration at cell–cell interfaces in both health and disease, as well as its relevance in numerous clinical applications, there is growing interest in platforms that facilitate mechanistic studies. Developing such platforms has been challenging due to the difficulty of precisely controlling the concentration and combination of ligands directly on the surface of cells. In this review, we first outline the challenges associated with manipulating ligands on cell surfaces, followed by a discussion of the available platforms that have enabled the study of signal integration along with their strengths and weaknesses.

## THE CHALLENGE OF MANIPULATING SURFACE LIGANDS DIRECTLY ON THE CELL SURFACE

The most widely used method to study the contribution of surface ligands to immune cell recognition is the use of genetically manipulated antigen presenting cells (APCs). These APCs can be immortal cell lines or primary cells isolated from blood or tissues, and various genetic methods are often used to manipulate the expression of specific ligands. By comparing the parental and genetically modified APCs, the contribution of specific ligands can be determined. This method has been widely used to study, for example, the mechanism of PD‐1 and BTLA co‐inhibition.^18^, ^19^, ^20^, ^21^

A limitation of using these conventional APCs is that they express different ligands, which means that different APCs will give different results. Moreover, ligands can be redundant. For example, genetic knockout of the adhesion ligands CD58 or ICAM‐1 in the U87 glioblastoma cell line produces a modest decrease in T‐cell antigen sensitivity because they both affect antigen sensitivity.^22^, ^23^ Lastly, many co‐signaling receptor/ligand interactions take place at the interface.^3^ As a result, the magnitude of the effect of manipulating a surface ligand will depend on the APC used.

**Figure 2:**
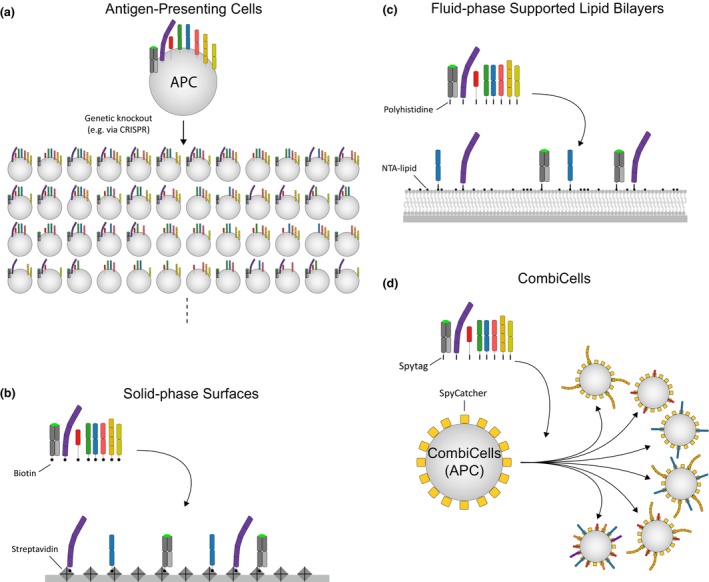
Platforms for displaying surface ligands to immune cells. **(a)** Genetically modified APCs express multiple surface proteins that act as ligands to receptors on immune cells. These proteins can be knocked out to generate cell lines with different ligand combinations, and transfections can be performed to alter ligand concentration. However, this requires the laborious generation and maintenance of multiple individual cell lines, which can diverge over time. In platforms that rely on **(b)** solid‐phase surfaces, **(c)** fluid‐phase SLB surfaces, and **(d)** CombiCells, the purified extracellular domain of ligands is coupled to each surface. As a result, it is straightforward to generate desired surfaces by adding different combinations and concentrations of each ligand. Solid‐phase surfaces commonly rely on attaching biotinylated ligands to streptavidin‐coated plastic or glass cell culture plates. Fluid‐phase SLBs commonly rely on attaching biotinylated ligands to biotinylated lipids through streptavidin or polyhistidine‐tagged ligands to NTA‐lipids. The CombiCell system involves a base cell line transfected to express a high surface concentration of the SpyCatcher protein, which forms a spontaneous covalent bond with a short peptide tag known as SpyTag. By adding desired combinations and concentrations of ligands fused to a SpyTag, cells can be generated displaying specific combinations and concentrations of surface ligands.

A potential solution to this problem is to generate APCs expressing different combinations of ligands (Figure 2a). APCs from very different species, such as insects,^24^ can be used to reduce the number of endogenous ligands. Unfortunately, the combinatorial complexity of this solution is large, making it impractical for studying more than 3–4 ligands. For example, generating APCs with all pairwise combinations of just 20 ligands would require nearly 200 cell lines. Moreover, generating cell lines often requires prolonged cultures that can lead to genetic drift between lines. This can introduce differences between lines other than those intended, requiring multiple lines to be developed independently to obtain robust results.^25^ Another limitation of this genetic approach is that the surface density of the ligands cannot be easily manipulated without creating even more cell lines.

## PLATFORMS THAT ENABLE PRECISE CONTROL OF LIGAND COMBINATION AND SURFACE DENSITY

The precise control of ligand combination and surface density has been achieved by using artificial surfaces. These can be solid‐phase, such as plastic or glass surfaces, or fluid‐phase, such as supported lipid bilayers (SLBs). In these platforms, the purified extracellular domains of receptor ligands are coupled to these surfaces at the desired combination and surface density (Figure 2b, c).

The simplest method is to absorb purified ligands onto a solid surface, such as plastic or glass (Figure 2b). For example, purified biotinylated pMHC antigens can be adsorbed onto streptavidin‐coated plastic or glass surfaces at different concentrations to assess the impact of antigen affinity and kinetics on T‐cell antigen sensitivity.^26^, ^27^, ^28^, ^29^ In such a system, it is trivial to add purified ligands along with pMHC, such as ligands for CD2, LFA‐1, CD28, CD27, 4‐1BB, OX40, and GITR, allowing exploration of their impact on antigen sensitivity and efficacy^22^, ^30^, ^31^ or on their ability to sustain cytokine production over time.^32^, ^33^, ^34^

An alternative method is to absorb purified ligands onto SLBs.^35^, ^36^ For example, purified ligands with a polyhistidine tag can be captured at different concentrations and combinations directly to bilayers containing lipids with an NTA head group. A key advantage of this method over solid‐phase surfaces is that the ligands are laterally mobile, as they are on cell surfaces. Initial SLB experiments focused on understanding how antigen concentration and/or affinity impacted T‐cell responses,^37^ while subsequent studies examined the impact of other receptor ligands on T‐cell activation, such as CD28, PD‐1, CD2, and LFA‐1.^18^, ^38^, ^39^, ^40^, ^41^

While fluid‐phase SLBs enable ligand mobility, it is difficult to produce SLBs in a high throughput format. Moreover, commonly used artificial surfaces are much more rigid than most cells, which is relevant given that mechanical forces play a critical role in immune recognition.^42^, ^43^ To address these shortcomings and the previously mentioned shortcomings of genetically modified APCs, we developed a new platform enabling combinatorial display of ligands directly on the cell surface (CombiCells)^44^ (Figure 2d). This platform relies on the engineered protein SpyCatcher, which forms a spontaneous covalent bond with a short peptide tag known as SpyTag.^45^ By expressing SpyCatcher on the cell surface, any combination and concentration of purified proteins engineered with a SpyTag can be coupled to the cell surface within minutes. The surface SpyCatcher is designed so that when the extracellular regions of proteins with a membrane‐proximal SpyTag are coupled to it, they resemble their native size. This was accomplished by fusing a truncated stalk containing 8 amino acids from CD52, which is a GPI‐anchored protein, to SpyCatcher in an orientation that minimizes any increase in size. We showed that surface SpyCatcher exhibits typical mobility of surface ligands and can be expressed in different cell lines, including CHO‐K1, U87, and Nalm6. We used the platform to study signal integration between the TCR and several co‐signaling receptors, including CD2, LFA‐1, CD28, and PD‐1, showing, for example, that PD‐1 can readily inhibit T‐cell activation by the TCR alone or in combination with CD2 or CD28 co‐stimulation.^44^

## CRITERIA FOR SELECTING SURFACE DISPLAY PLATFORM

While all four platforms are useful for studies of T‐cell recognition, they have distinct strengths and weaknesses that make them particularly suitable for studying different aspects of how co‐signaling receptor/ligand interactions impact immune cell responses (Table 1).

**Table 1:** Strengths and weaknesses of ligand display platforms.^a^

	Conventional APCs	Solid‐phase surface	Fluid‐phase SLB surface	CombiCells
Can ligand combinations be easily tested?	×	✓	✓	✓
Can ligand concentrations be easily tested?	×	✓	✓	✓
Is absolute ligand size conserved?	✓	×	✓	×
Is relative ligand size conserved?	✓	✓	✓	✓
Is ligand mobility conserved?	✓	×	×	×
Can ligand spacing be controlled?	×	×	✓	×
Is surface stiffness conserved?	✓	×	×	✓
Is glycocalyx conserved?	✓	×	×	✓
Is surface feedback conserved?	✓	×	×	×
Is platform high‐throughput?	×	✓	×	✓

^a^ We have indicated ✓ or × for each platform based on how the platform is commonly used (see text for details).

### Ligand combinations and concentrations

T cells interact with a wide variety of normal cells as well as cancerous and infected cells. As a result, they are exposed to a wide range of ligand combinations and concentrations. The importance of studying ligand combinations has been highlighted by the suggestion that PD‐1 inhibits CD28 costimulation rather than TCR signaling.^18^ It follows that the effect of the engagement of one ligand may depend on the expression of other ligands. Such effects can only be explored by systematically varying ligand combinations and surface densities.

This high level of diversity can easily be reconstituted by the addition of purified ligands at any concentration and combination in the solid‐ and fluid‐phase surfaces, and in the CombiCell platform. This made it easy to study T‐cell activation in response to CombiCells displaying 12 surface densities of antigen alone or together with pairwise combinations of ligands to CD28, CD2, and PD‐1, comprising 72 independent conditions. This revealed that PD‐1 can inhibit T‐cell responses to antigen alone as well as with CD28 and CD2 co‐stimulation.^44^

In contrast, using genetic methods to produce many conventional APCs with different combinations or concentrations of ligands is impractical. In part, this is because it requires a large number of cell lines to be produced. It also requires cell sorting and prolonged culture to establish desired panels of cell lines, which may lead to genetic drift resulting in undesired differences between lines. Moreover, the low sensitivity of flow cytometry makes it difficult to sort cells with very low ligand surface densities. This is particularly important for T cells, which can recognize as few as 1–100 antigen ligands on their target cells.^46^, ^47^ By titration of antigen on CombiCells, we have shown T cells can be fully activated when antigen is undetectable by flow cytometry.^44^

### Absolute and relative ligand size

The extracellular size of receptor/ligand interactions at immune cell interfaces is known to vary widely^48^, ^49^ and to be functionally important.^39^, ^50^, ^51^, ^52^, ^53^, ^54^

The absolute intermembrane distance spanned by immunoreceptor/ligand interactions is a critical feature of the kinetic‐segregation (KS) model of immunoreceptor triggering.^55^, ^56^ The KS model postulates that TCRs and other immunoreceptors transduce signals when ligand engagement traps them in close contacts from which tyrosine phosphatases, such as CD45 and CD148, are excluded because of their large extracellular domains.^57^ In support of this, elongation of the TCR/pMHC interaction, which spans an intermembrane distance of ∼14 nm,^58^ reduces T‐cell responses to pMHC.^50^, ^51^ The relative intermembrane distance spanned by different receptor/ligand complexes is also important for signal integration. For example, elongation of the CD2/ligand complex, which also spans ∼14 nm,^59^, ^60^ impairs T‐cell sensitivity to pMHC, presumably by positioning the membranes too far apart for TCRs to efficiently engage pMHC.^52^, ^54^ Matched receptor/ligand dimensions have been shown to be important for signal integration between activatory and inhibitory immunoreceptors.^39^, ^53^, ^54^ Thus, ligand size is functionally important for immune cell recognition.

Although absolute ligand size is only entirely conserved with conventional APCs expressing native ligands, the use of oligo‐histidine‐tagged ligands coupled to NTA lipids is expected to approximate ligand size in SLBs. In contrast, the relative ligand size is preserved in all platforms as long as the ligands are coupled using the same method. With CombiCells, all ligands are covalently coupled to surface SpyCatcher, which is expected to increase the physical size of all coupled ligands by up to 4 nm.^44^ Coupling biotinylated ligands via streptavidin to coated glass/plastic or SLBs increases their absolute size by 6–8 nm. Thus, while all platforms can be used to study processes where only relative size is important, genetically modified APCs and SLBs are preferable where absolute size may be important.

### Ligand mobility

The ligands on APCs, like all cell surface molecules, are mobile and diffuse in the plane of the membrane. Their mobility and distribution depend on a number of factors. These include the lipid composition of the membrane, interactions with the underlying cytoskeleton, the presence of other membrane‐anchored molecules, and engagement of receptors on immune cells.^61^, ^62^, ^63^ For example, upon TCR engagement the diffusion of pMHC antigens in SLBs slows dramatically.^64^ Subsequently, pMHC microclusters form (∼200–400 nm), and these are then transported centripetally to form a large (∼2000–4000 nm) central supramolecular activation cluster or cSMAC.^37^, ^65^ When multiple ligands are presented on the SLB surface, they redistribute in the contact interface according to size: smaller receptor/ligand interactions co‐localize with TCR/pMHC complexes (e.g. CD28/CD80, PD‐1/PD‐L1, CD2/CD58) and larger receptor/ligand interactions (e.g. LFA‐1/ICAM‐1) and larger molecules (e.g. CD45) segregating from TCR/pMHC complexes.^18^, ^39^, ^40^, ^65^ Broadly similar rearrangements are observed when T cells engage APCs.^66^

With solid‐phase platforms, processes that depend on spatial redistribution, such as clustering and colocalisation, will be impaired because ligands are immobile. In contrast, spatial redistribution is enabled in SLBs and CombiCells because they rely on lipid anchored ligands that remain mobile with diffusion coefficients similar to ligands on the cell surface.^36^, ^44^ In the SLB platform, mobility can also be varied by changing the bilayer lipid composition from higher mobility 1‐palmitoyl‐2‐oleoyl‐glycero3‐phosphocholine (DOPC) to lower mobility 1,2‐dipalmitoyl‐sn‐glycero‐3‐phosphocholine (DPPC), which forms a gel‐phase SLB.^67^, ^68^ It is also possible to maintain mobility for some ligands and immobilize others by fabricating ‘micro‐dots’ of immobile ligands surrounded by a traditional fluid‐phase SLB.^69^

We note that ligand mobility along with extracellular size‐matching is likely to be required for certain mechanisms of signal integration,^57^ including the direct dephosphorylation of the cytoplasmic tails and/or proteins associated with the cytoplasmic tails of the TCR‐CD3 complex and CD28 by the inhibitory receptors BTLA and PD‐1, respectively.^18^, ^39^ We have previously estimated that PD‐1 must be within ∼13 nm of CD28 for its associated phosphatase to reach its substrate.^70^

While fluid‐phase SLBs and CombiCells allow for ligand mobility, this mobility is uniform for all coupled ligands. In contrast, ligands that are natively expressed on APCs can have different mobilities because mobility depends on their transmembrane and cytoplasmic domains. For example, the cytoskeleton has been shown to regulate the mobility of the ligand ICAM‐1 but not MHC‐II.^71^ It is possible to maintain mobility for some ligands and immobilize others by fabricating ‘micro‐dots’ of immobile ligands surrounded by a traditional fluid‐phase SLB.^69^ While this introduces two ligand mobility classes (mobile or immobile), conventional APCs may be better suited for investigating how diverse ligand‐specific mobilities impact ligand function.

### Ligand spacing

Spatial patterns have been documented to form at immune cell interfaces,^37^, ^65^, ^66^, ^72^ but their functional importance is often unclear.^73^ Imposing spatial patterns provides a mechanism to explore their functional importance. For example, it has been hypothesized that membrane‐proximal signal integration between immunoreceptors requires nanometer‐scale co‐localisation.^39^, ^53^, ^54^, ^57^ A number of studies have identified mechanisms consistent with this hypothesis, including the direct dephosphorylation of the TCR, CD28, and other membrane‐proximal substrates by the tyrosine phosphatases SHP‐1/SHP‐2 when associated with the co‐inhibitory receptors BTLA and PD‐1.^18^, ^21^

When solid‐phase surfaces are used, a variety of methods are available to control ligand spacing. For example, microcontact printing/fabrication enables patterns of proteins to be coupled to surfaces.^74^, ^75^, ^76^, ^77^, ^78^, ^79^, ^80^, ^81^ A limitation of these methods is their spatial resolution. To enable nanometer‐scale control, DNA‐based origami platforms have been developed, which can be used on solid‐phase or fluid‐phase SLB surfaces.^82^, ^83^ In these platforms, biotinylated ligands can be immobilized at precise locations to biotinylated ‘staple’ strands on the rectangular origami platforms using divalent streptavidin.^82^ Such platforms revealed that a single native ligand (pMHC) can activate T cells while two artificial ligands (CD3‐binding Fabs) in close proximity are needed.^82^ A recent study has shown that origami platforms can be directly coupled to cell surfaces,^84^ raising the possibility that ligand spacing can be controlled directly on the cell surface. In this case, it is important to note that ligands presented on the origami platform will be expected to display different extracellular size and mobility, and may impact the ability of ligands to co‐localize.

Studies that present some ligands on origami platforms typically present other ligands directly on the underlying substrate. This is also the case when coupling platforms directly to cell surfaces, as these have many native ligands. A drawback of these approaches is that they introduce artificial differences in extracellular ligand size, which may disrupt signaling by, for example, impeding co‐clustering.

### Surface stiffness

T cells and other immune cells interact with a wide range of cells and particles that vary enormously in surface stiffness.^42^, ^85^, ^86^, ^87^ While the mechanical properties of cells vary, introducing such variation, e.g. by using different cells, usually varies other factors, making it challenging to isolate the effects of surface stiffness on immune cell activation.

To overcome this limitation, artificial solid‐phase surfaces with different mechanical properties have been developed.^86^, ^88^, ^89^, ^90^ For example, streptavidin can be embedded in poly‐acrylamide gels with desired surface rigidities to immobilize any concentration and combination of biotinylated ligands.^86^ A further advantage of using soft gels with well‐defined properties is that imaging can be used to quantify local deformation generated by cellular forces. For example, traction force microscopy^91^ has revealed that T cells can generate large ∼150 pN forces.^92^ Another novel way to assess mechanical forces is to engineer the topography of solid‐phase surfaces with micropillars whose deformation by immune cells can be quantitatively assessed to determine the mechanical abilities of immune cells.^81^, ^93^

Fluid‐phase SLBs are most commonly supported by a glass surface whose stiffness, like conventional solid‐phase surfaces, is many orders of magnitude higher than conventional APCs that immune cells would encounter.^85^ To address this, a recent method has been developed to attach bilayers to softer polydimethylsiloxane (PDMS), revealing that late but not early steps in T‐cell activation are sensitive to surface stiffness.^94^ Similarly, soft sheets of plasma membrane can be generated directly from cells and decorated with ligands. Using such an approach, it was shown that B cells extract antigens more easily from softer surfaces.^95^

In summary, while conventional APCs and CombiCells conserve the surface stiffness of the underlying cell that is being used, it is challenging to use these platforms to study how variation in surface stiffness impacts immune cell responses. Platforms based on solid‐phase and fluid‐phase surfaces can be generated with defined mechanical properties while preserving other aspects, such as ligand mobility and size. This enables the study of how mechanical factors influence immune cell activation.

### Surface Glycocalyx

All cell surfaces, including immune cells^96^ and cancer cells^97^ have large glycoproteins and proteoglycans on their surfaces, forming a thick (> 50 nm) glycocalyx. While most immunoreceptors and their ligands are short (spanning ≈ 14 nm when bound), immune cells have many large (> 30 nm) and abundant glycoproteins (e.g. CD43, CD45 and CD148).^57^ The glycocalyces of immune cells and other cells, especially cancer cells, form a barrier that must be overcome to enable the close intermembrane contacts to form that are essential for immunoreceptors to engage their ligands on APCs. These close contacts are likely to be formed by microvilli‐like structures, formed by actin polymerization.^98^ For example, T cells have been shown to use microvilli‐like protrusions to bring their membranes into the ≈14 nm close contact required for the TCR to bind pMHC.^58^, ^99^, ^100^, ^101^

Although present in APCs and CombiCells, glycocalyx proteins are generally omitted from artificial surfaces. A recent study incorporated the large extracellular domains of CD45 and CD43 in a SLB to reproduce a basic glycocalyx.^102^ This enabled dynamic visualization of microvilli‐generated close contact formation by monitoring the exclusion of CD45 and CD43, confirming CD2 engagement of CD58 stabilized close contacts where they co‐localized with TCR/pMHC complexes.^102^ Unlike APC and CombiCells, which natively include a glycocalyx, the SLB platform enables the glycocalyx to be readily manipulated.

### Surface feedback

When immune cells interact with their target APC, the surface of the APC is expected to undergo dynamic passive and active energy‐consuming processes on multiple spatial and temporal scales.^103^, ^104^, ^105^, ^106^ For example, dendritic cells dynamically reorganize their cytoskeleton in response to contact with T cells, and this feedback process ultimately contributes to the magnitude of T‐cell activation.^103^ Similarly, cancer cells can dynamically stiffen, soften, and polarize their cytoskeleton upon contact with T cells, and these changes impact the ability of T cells to kill them.^105^

Conventional APCs is the only platform that can fully recapitulate bi‐directional surface feedback. As solid‐phase and fluid‐phase SLB surfaces lack an underlying cellular scaffold, and hence an underlying cytoskeleton, they are unable to recapitulate active energy‐consuming processes such as dynamic changes in surface stiffness. The use of a cellular scaffold in the CombiCell platform means that some generic elements of feedback are likely preserved, but this has yet to be investigated. It is important to note that because all ligands are coupled to the same generic surface SpyCatcher molecule, any signaling‐induced feedback in the CombiCell platform will uniformly apply to all ligands, whereas signaling‐induced feedback on conventional APCs can be ligand‐specific depending on the intracellular signaling domain of the ligand (see also Ligand Mobility).

### Experimental throughput and surface stability

Platform throughput can be critical when studying how large combinations and concentrations of ligands control immune cell decisions. The solid‐phase assay and CombiCells achieve very high throughput by using the 96‐well format.^30^, ^44^ APCs are impractical because new APC lines are required for each ligand combination and concentration. While easy to manipulate, the fragility of SLBs, the challenge of cleaning glass substrates in the 96‐well format, and a reliance on imaging techniques have limited the throughput of SLB studies to ∼10 conditions per day, although efforts are underway to improve throughput.^107^

One drawback of CombiCells is that its ligands are lost from the cell surface over time as a result of endocytosis and degradation of the ligand‐SpyCatcher complex. This process is cell‐type specific with a lifetime of ≈9 h on CHO‐K1 cells and more than ≈24 h on Nalm6 cells, for example.^44^ It should be noted that other systems encounter similar drawbacks, as ligands can be degraded or unfold over this time scale. In the case of APCs, ligand stability should be longer, as they are able to continuously synthesize new ligand. However, ligand levels can vary during longer experiments because of changes in expression and turnover and processes such as transendocytosis.^108^

## CONCLUSION

The study of signal integration at immune cell interfaces is now entering an exciting phase where many platforms can readily be used to understand cellular activation decisions. Despite the large number of receptor/ligand interactions that contribute to any immune cell interaction, the platforms available to date have limited our ability to understand how these interactions cooperate to generate responses in immune cells. These platforms have either been too low throughput (genetically modified APCs and SLBs) or lacked essential properties such as ligand mobility (solid‐phase surfaces). The availability of CombiCells, which allow many combinations and surface densities to be rapidly tested, will help address this technology gap. However, other techniques have specific advantages over CombiCells, such as better conservation of absolute ligand size and control of ligand spacing (Table 1). By understanding the strengths and weaknesses of each platform, including which factors can be controlled by the user, immunologists can identify the most appropriate platform to address their hypothesis. It should be stressed that, in addition to receptor/ligand interactions at the cell–cell interface, the outcome of cellular interactions also depends on many other factors, such as the differentiation state of the cell and the availability of soluble ligands such as cytokines. The combinatorial interplay between cell state, soluble ligands, and ligands displayed on other cells will require novel platform innovations for systematic explorations of immune cell activation.

## CONFLICT OF INTEREST

P Anton van der Merwe and Omer Dushek have financial interests in a filed patent application related to CombiCells (WO2024231680A1).

## AUTHOR CONTRIBUTIONS

**Jordan Kramer:** Conceptualization; investigation; visualization; writing – original draft; writing – review and editing. **P Anton van der Merwe:** Conceptualization; investigation; project administration; supervision; writing – original draft; writing – review and editing. **Omer Dushek:** Conceptualization; funding acquisition; investigation; project administration; supervision; visualization; writing – original draft; writing – review and editing.
